# Supplementary material for: GCR1 Positively Regulates UV-B- and Ethylene-Induced Stomatal Closure via Activating GPA1-Dependent ROS and NO Production
Source: Int J Mol Sci. 2022 May 15;23(10):5512. doi: 10.3390/ijms23105512 (PMC9141438; doi:10.3390/ijms23105512)
Supplement: Supplementary file 1 [file ijms-23-05512-s001.zip › ijms-1703221-supplementary.pdf]

Supplementary materials

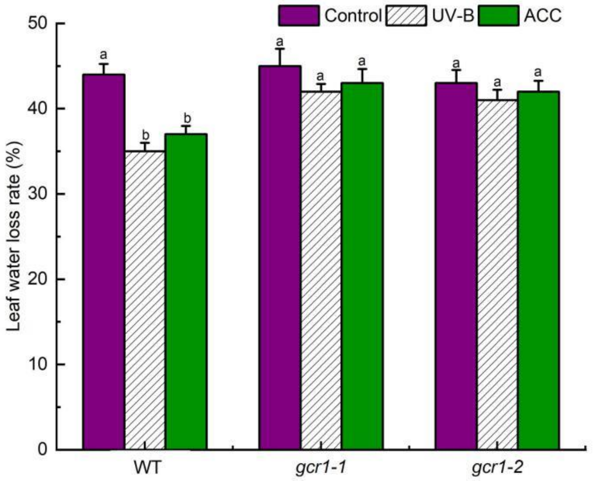

Figure S1. Effect of UV-B and ACC on the leaf water loss rate in wild-type Col-0 (WT) and *gcr1* mutants. The leaf water loss rate was calculated after leaves pretreated with MES buffer alone or with 100 μmol/L ACC (ACC) under white light (0.1 mmol/m^2/s) alone or supplemented with 0.5 W/m^2 UV-B radiation (UV-B) for 3 h were placed under light without MES buffer for 2 h. Data are shown as the means ± SE of ten leaves.

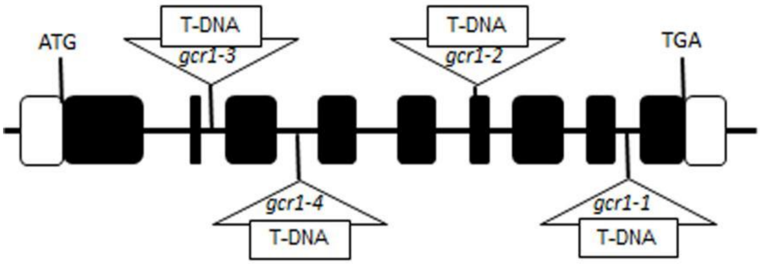

Figure S2. T-DNA insertion mutant alleles of GCR1 in Arabidopsis. Boxes and lines represent exons and introns, respectively (figure not drawn to the scale).

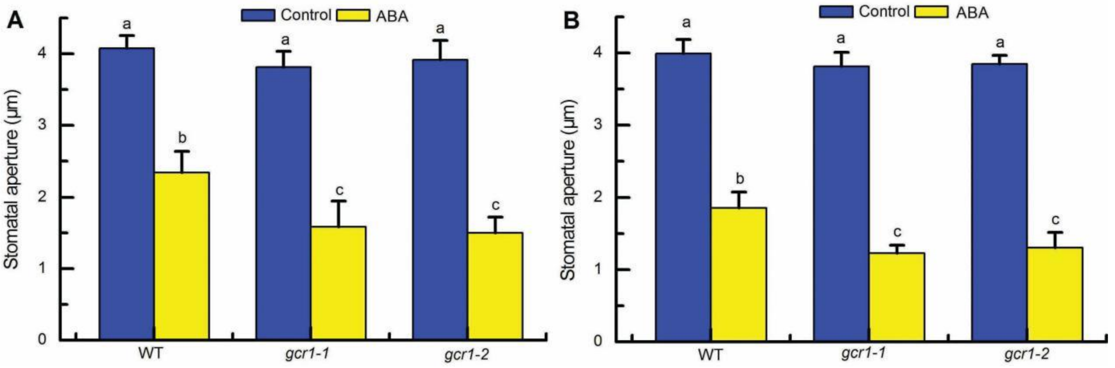

Figure S3. GCR1 plays a negative role in ABA inhibition of stomatal opening and promoting of stomatal closure. (**A**) Leaves of the WT and *gcr1* mutants were incubated in MES buffer under darkness for 3 h to close the stomata, and then incubated in MES buffer alone (Control) or containing 10 μmol/L ABA (ABA) for 3 h under the light. (**B**) Leaves of the WT and *gcr1* mutants with open stomata were incubated in MES buffer alone (Control) or containing 10 μmol/L ABA (ABA) for 3 h under the light. After treatments, stomatal apertures were measured in epidermal strips taken from the abaxial surfaces of the treated

leaves. Data are presented as means ± SE of three independent experiments, each with 30 stomata. Means with different letters are significantly different at $p < 0.01$.
